# Towards Computational Screening for New Energetic Molecules: Calculation of Heat of Formation and Determination of Bond Strengths by Local Mode Analysis

**DOI:** 10.3389/fchem.2021.726357

**Published:** 2021-07-20

**Authors:** Imogen L. Christopher, Adam A. L. Michalchuk, Colin R. Pulham, Carole A. Morrison

**Affiliations:** ^1^ EaStCHEM School of Chemistry, University of Edinburgh, Edinburgh, United Kingdom; ^2^ Federal Institute for Materials Research and Testing (BAM), Berlin, Germany

**Keywords:** heat of formation, computational screening, lattice energy calculations, local force constants, energetic materials

## Abstract

The reliable determination of gas-phase and solid-state heats of formation are important considerations in energetic materials research. Herein, the ability of PM7 to calculate the gas-phase heats of formation for CNHO-only and inorganic compounds has been critically evaluated, and for the former, comparisons drawn with isodesmic equations and atom equivalence methods. Routes to obtain solid-state heats of formation for a range of single-component molecular solids, salts, and co-crystals were also evaluated. Finally, local vibrational mode analysis has been used to calculate bond length/force constant curves for seven different chemical bonds occurring in CHNO-containing molecules, which allow for rapid identification of the weakest bond, opening up great potential to rationalise decomposition pathways. Both metrics are important tools in rationalising the design of new energetic materials through computational screening processes.

## Introduction

Energetic materials (explosives, propellants, pyrotechnics and gas generators, EMs) are characterised by their ability to rapidly convert chemical potential energy into kinetic energy. Safety is of paramount importance in this field and takes precedence over performance, such that only a limited number of EMs have found employment in civilian and military applications. Many of the well-established EMs, however, do not comply with increasing environmental and public health regulations (Cumming, 2017), which fuels the need to develop new non-toxic and environmentally benign EMs that do not compromise on safety or performance metrics.

Computational screening presents an attractive route in the new materials pipeline, as it offers a cost-effective way to assess candidates prior to synthesis. This is particularly desirable in the field of EMs where safety testing (such as impact, spark and friction sensitivity measurements) typically require gram-scale quantities of compounds to be synthesised. This is potentially very hazardous work when the material is novel and may initiate on the slightest perturbation.

Having access to reliable predictive models also opens up routes to the rational design of new EMs, by offering a path towards understanding structure-property relationships. Previous work in our group has focused on the ability to predict the impact sensitivities of EMs using first principles simulations, and our methods, which are based on a vibrational up-pumping model, have demonstrated success for a range of structurally diverse materials that exhibit widely varying sensitivities to initiation ( Michalchuk et al., 2018a; Michalchuk et al., 2019; Michalchuk et al., 2021). This predictive model highlighted the importance of low energy (*ca*. 200 ± 50 to 600 ± 150 cm^−1^) molecular vibrational modes to channel (up-pump) the energy arising from the phonon scattering of the many low energy lattice mode vibrations which become vibrationally hot following a mechanically-induced shock event. Trapping this energy in the low energy molecular vibrations induces large amplitude vibrations that distort the molecular structure to the degree that electronic changes occur: band gaps narrow, electrons flow, and unstable species emerge all on the timescale of a molecular vibration (Michalchuk et al., 2018b). This marks the start of initiation. It therefore follows that crystal lattices with a high density of low-lying molecular vibrations will likely be sensitive to shock and impact-induced initiation. The reverse also holds true: crystal lattices that are vibrationally sparse in this region will likely be shock and impact-insensitive, and are thus safer to handle.

Predicting impact sensitivities is only one aspect of an EM computational screening process. Also of key importance is the stored energy in the molecule, which can be gleaned from the solid-state heat of formation energy, ∆H_f(s)_. This provides a route to calculate the heat of combustion, which in turn allows prediction of several parameters including the detonation pressure and the velocity and heat of explosion (Politzer et al., 2003), using thermo-chemical software methods such as CHEETAH (Fried and Souers, 1994) or EXPLO-5 (Suceska, 2004).

Multiple routes to calculating ∆H_f(s)_ have been previously proposed in the literature. Our purpose here is to signpost the current state of the art, and to show its application to EMs. The first step in calculating ∆H_f(s)_ begins with the gas-phase heat of formation, ∆H_f(g)_, and various methods have been proposed for this over the years. For instance, Benson’s group increment theory (BIGT) exploited experimental heats of formation for individual groups of atoms to develop group equivalence values for linear and branched alkanes and alkenes (Benson and Buss, 1958). While impressive at the time of inception, its application is limited to the types of molecules represented in the training set, and so it has limited scope beyond this area. More recently, quantitative structure-property relationship (QSPR) models have been realised as a powerful tool to explore the relationships that link molecular structure to material properties. Vatani et al. (2007) devised a new QSPR method for predicting heats of formation for over 1,000 organic molecules, covering almost all organic functional groups. However, transition-metal and main-group elements were not included in the training set, so whilst excellent results were obtained, widespread application is again limited.

Within the field of EMs isodesmic reaction equations (Hehre et al., 1970; Ponomarev and Takhistov, 1997) and the atom- and group-equivalence methods (Byrd and Rice, 2006) are commonly applied. The former relies on a reaction equation where the types of chemical bonds in the reactants and products are conserved, and the heats of formation are known for all other molecules in the reaction except the one unknown (Cramer, 2004). Any intrinsic error associated with calculating any particular chemical bond is thereby cancelled out, meaning that relatively low-level computational methods can be employed to give fairly accurate results (Hehre et al., 1970). This method has been employed in computational chemistry for over 50 years, with recent developments automating the generation of parts of the isodesmic reaction equation (Chan et al., 2020). However, as a general technique it has found little application beyond CHNO-containing molecules.

The atom-equivalence method developed by Byrd and Rice is an advance on BGIT and has been extensively applied to EMs (Singh et al., 2014a; Singh et al., 2014b; Axthammer et al., 2015; Piercey et al., 2016), but is again restricted to CHNO-containing molecules. Here atom equivalence energy values for the four atoms were determined through comparison of experimental heats of formation for molecules in a training set and their computationally derived molecular energies (Byrd and Rice, 2006). This method works well, reporting root mean square deviation of 12.6 kJ mol^−1^ and is arguably more efficient than the isodesmic equation route, as it requires just the optimised energy of the molecule of interest expressed at a prescribed quantum mechanical model chemistry. A further advantage is the absence of any reliance on further experimental data. However, the continued application to CHNO-only molecules limits its application in a computational screening programme for new EMs which should have the flexibility to draw upon main-group and transition-metal elements.

Given the limitations of these two methods, herein we have pursued the use of PM7 to determine ∆H_f(g)_ (Stewart, 2013). This has been shown by Wan et al. to out-perform previous semi-empirical methods for a set of 142 organic molecules (Wan et al., 2020). Elioff et al. evaluated its capabilities compared to both the isodesmic and the group equivalence methods for nitrogen-containing organic molecules (Elioff et al., 2016). While the outcome showed that PM7 was the least accurate of the three (*R* ^*2*^ line of best fit against experimental data = 0.986, compared to 0.999 and 0.995 for the isodesmic and atom equivalence methods, respectively), it still performs relatively well, and has the advantage of being an easily deployed method that can be used for any molecule containing atoms from H−La and Lu−Bi. Fomin et al. (2017) tested the PM7 method alongside other semi-empirical methods, namely PM6, PM5, PM3, AM/1, and MNDO, for calculating ∆H_f(g)_ for copper and alkaline Earth metal complexes. They concluded that PM6 and PM7 both perform well, reporting *R* ^*2*^ values of 0.961 and 0.960, respectively. While these results are encouraging, further validation for the accurate prediction of ∆H_f(g)_ for a broader range of inorganic molecules would be welcome and will be provided here. The PM7 method also carries the advantage that no further calculations beyond a geometry optimisation are required, which renders it attractive as part of a high throughput study. Moreover, semi-empirical calculation methods have a wide application and user base, and are being continuously improved (Wan et al., 2020).

Converting ∆H_f(g)_ to ∆H_f(s)_ requires the addition of an intermolecular interaction energy term, as captured by the sublimation energy, ∆H_sub_, or the lattice energy, ∆H_L_ (formally, ∆H_L_ = −∆H_sub_ – 2RT). For single-component molecular solids, Politzer et al. have developed a route to determine ∆H_sub_ from consideration of the electrostatic potential (ESP) (Politzer et al., 1997; Politzer et al., 1998). While they have applied this analysis to estimate many liquid, solid, and solution parameters dependent on non-covalent interactions (Politzer and Murray, 1998; Politzer and Murray, 2002) it is their relationship to calculate ∆H_sub_ which is most relevant here. The method was developed initially using a training set of 34 CHNO-containing molecules, and further parametrised by Byrd et al. using a training set of 38 CHNO-containing energetic molecules (Byrd and Rice, 2006). The ESP method tested here uses the parameters proposed by Byrd, as parameterisation was carried out at using the B3LYP/6-31G* computational model, rather than the HF/STO-5G* level originally used by Politzer. In addition, Byrd’s training set included functional groups more likely to be present in EMs (e.g., azides and nitro groups).

For salts, an attractive route to ∆H_f(s)_ from ∆H_f(g)_ is *via* calculation of ∆H_L_ using the method developed by Jenkins et al. (1999), Jenkins et al. (2002) that relies only on knowledge of the molecular (formula unit) volume and the stoichiometry of the salt. This method has been used by Gao et al. (2007) to compare the estimated values of ∆H_f(s)_ for 33 energetic salts to their respective experimental values, using the G2 method to calculate the ∆H_f(g)_ terms for the ions based on their proton or electron affinities. They reported *R* ^*2*^ = 0.983, with a maximum deviation of 158.5 kJ mol^−1^.

Co-crystals are an important development in the field of EMs (Kennedy and Pulham, 2018). While acknowledging that predicting whether or not a stable co-crystal will form from its single-component species is in itself the greater challenge, having the ability to predict ∆H_f(s)_ for known materials is also important. This is particularly true for EM research, where creating materials that can store large amounts of chemical energy is an essential requirement. Previous attempts have met with limited success. For instance, Zhang et al. (2016), Bozkuş et al. (2019) both used an atomisation energy method (Curtiss et al., 1997) which can formally only be used to calculate the heat of formation of gas-phase species. Ma et al. (2017) used isodesmic equations to calculate ∆H_f(g)_ for the co-formers of a CL-20/MTNP co-crystal, and then predicted ∆H_sub_ using a relationship based on the melting point of the co-crystal. While this method is promising, its application is limited if the melting point is not known. Gavezzotti developed the PIXEL method to calculate ∆H_L_ as a sum of Coulombic, polarisation, dispersion and repulsion intermolecular energies in a crystal structure and has been shown to have the correlation *R* ^2^ = 0.845 between experimental and calculated ∆H_L_ for 154 organic crystal structures (Gavezzotti, 2011). The method was recently expanded to include parameterisation for transition metal complexes (Maloney et al., 2015). Another route to obtaining ∆H_L_ is through the more computationally demanding dispersion-corrected density functional theory (DFT-D) (Morrison and Siddick, 2003; Feng and Li, 2006; Brandenburg et al., 2015; Fan et al., 2017). For the specific example of co-crystals considered here, as it is uncommon to measure ∆H_f(s)_ we shall compute formation enthalpies using both PIXEL and DFT-D for direct comparison.

In addition to having reliable routes to predict ∆H_f(s)_, having knowledge of the strengths of the individual bonds within a molecule is also valuable information at the EM molecular design stage, as it can provide information on the first-stage initiation pathway. Historically, intramolecular bond strengths have been calculated through heterolytic bond cleavage reactions, but this proves problematic beyond the first bond breaking reaction, as each subsequent bond breakage reaction is performed on increasingly unstable molecular fragments (Cremer et al., 2000), or requires a separate bond breaking reaction for each bond to be investigated (Zou et al., 2012). This is particularly problematic for ring systems, where breaking one bond introduces additional strain in the remaining bonds, such that isolating one bond becomes an impossible task. Alternatively, computation of the bond force constants offers a direct route to determining the bond strengths of all bonds within a molecule without recourse to molecular fragmentation. However, the normal modes of molecular vibration, from which the force constants are extracted, are commonly a complex mix of bond stretching, angle bending and twisting motions, meaning that pure bond force constants can rarely be obtained. Recent developments by Konkoli and Cremer (Konkoli and Cremer, 1998a; Konkoli and Cremer, 1998b; Kraka et al., 2020) have allowed for the mass decoupling of the normal modes of vibration, to recast the eigenvectors onto a new set of modes, termed the local modes of vibration, that correlate directly with individual bond stretches, angle bends etc. Their work has shown that the resulting local-mode force constants thus obtained are a direct measure of bond strength (Kraka and Cremer, 2019). Thus performing local mode analysis across a broad range of molecules (both energetic and non-energetic) provides information on the relationship between bond length and bond strength of the most common chemical bonds in EMs, which has the potential to be utilised for molecular design.

Herein, a set of 20 CHNO-containing molecules and a further 31 inorganic molecules was constructed to benchmark the PM7 method against isodesmic equation reactions and the atom equivalence method to calculate ∆H_f(g)_. Additionally, methods for converting ∆H_f(g)_ to ∆H_f(s)_ using the methods proposed by Byrd, Jenkins, and by the PIXEL method/DFT-D for single-component molecular crystals, salts and co-crystals, respectively, were also pursued for 48 compounds. Local vibrational mode analysis has also been carried out on 30 molecules containing chemical bonds found in energetic molecules, to evaluate bond length/strength relationships and to ascertain the likely weakest bonds in energetic molecules. Finally, both parameters have been highlighted for their potential to be included in a computational screening program for new energetic materials.

## Computational Methods

All optimization and vibrational frequency calculations were performed at the B3LYP/6-31G* level, as implemented in Gaussian16 (Frisch et al., 2016).

### Gas-Phase Heat of Formation: Isodesmic Equations

Equations were devised to ensure the type of chemical bonds were conserved, and that the heats of formation of all other molecules in the equation were known (see Supplementary Table 2). The heat of reaction, *∆H*_*R*_, is then calculated according to Eq. 1.

$$\Delta H_{R}=\Delta E_{0}+\Delta \mathrm{ZPE}+\Delta H_{T}+\Delta \mathrm{nRT}$$ (1)

Where *ΔE*_*0*_ is the change in energy between the products and reactants, *ΔZPE* is the change in the zero-point energies between products and reactants, and *ΔH*_*T*_ is the thermal correction from 0 to 298 K. As the number of atoms remain constant in the reaction, *ΔnRT* equals zero. The calculated heat of reaction is then equated to Eq. 2. Assuming the molecule of interest is a reactant in the isodesmic equation then its heat of formation is calculated by subtracting the known heats of formation of the other reactants and products from ∆H_R._

$$\Delta H_{R}=\sum \Delta H_{f\left(g\right)products}\;+\sum \Delta H_{f\left(g\right)reactants}\;$$ (2)

### Gas-Phase Heat of Formation: Atom Equivalence Method

Formation energies were determined according to Eq. 3, where *E* is the optimised energy of the molecule, *n*_*j*_ is the number of atoms of type *j* and *e*_*j*_ is the atom equivalence value of atom *j*, as determined by Byrd and Rice (Byrd and Rice, 2006).

$$\Delta H_{f\left(g\right)}=E-\sum n_{j}e_{j}\;$$ (3)

### Gas-Phase Heat of Formation: PM7

PM7 (Stewart, 2013) method was utilised as presented in Gaussian 16, using geometries optimised to global minima from the previously mentioned calculations for improved accuracy. For molecules containing third row and higher atoms, the SCF = YQC algorithm was used, as suggested in the PM7 documentation.

### Solid-State Heat of Formation: Single-Component Solids

The solid heat of formation for single component materials were calculated using Eq. 4, where ∆H_f(g)_ was calculated using PM7 as described above, and ∆H_sub_ was calculated using the ESP method as described by Eq. 5.

$$\Delta H_{f\left(s\right)}\;=\Delta H_{f\left(g\right)}\;-\text{Δ}H_{sub}$$ (4)

$$\text{Δ}H_{sub}=a{\left(SA\right)}^{2}+b\sqrt{\sigma_{tot}^{2}\nu }\;+c$$ (5)

Where a, b and c are semi-empirically deduced fitting parameters proposed by Byrd et al*.* (Byrd and Rice, 2006) SA is the surface area of the 0.001 electron.bohr^−3^ isosurface of the electrostatic potential of the molecule, $\sigma_{tot}^{2}\;$is the measure of variablity of electronic potential on the surface, and ν is the degree of balance between the positive and negative charges on the isosurface. The latter three parameters were calculated using Multiwfn (Lu and Chen, 2012).

### Solid-State Heat of Formation: Salts

For the energetic salts ΔH_f(s)_ was calculated from Eq. 6, where ∆H_f(g)_ of the cations and anions were calculated using PM7 as described above.

$$\Delta H_{f\left(s\right)}\;=\;\Delta H_{f\left(g\right)\left(cation\right)}+\;\Delta H_{f\left(g\right)\left(anion\right)}-\;\Delta H_{L}$$ (6)

Here, ∆H_L_ is expressed by Eq. 7, as proposed by Jenkins et al. (2002).

$$\Delta H_{L}=U_{pot}+\left[p\left(n_{m}/2-2\right)+q\left(n_{x\;}/2-2\right)\right]RT$$ (7)

Where $n_{m}$ and $n_{x\;}$ are constants that depend on the nature of the ions, and are set to 3 for monoatomic ions, 5 for linear polyatomic ions and 6 for non-linear polyatomic ions. The variables p and q denote the relative charges of the respective ions. The term $U_{pot}$ denotes the lattice potential energy and in turn is defined by Eq. 8.

$$U_{pot}=\alpha {\left(V_{m}\right)}^{\frac{1}{3}}+\beta$$ (8)

Where $V_{m}\;$denotes the molecular volume (V_cell_/*Z*), in nm^3^, and the remaining coefficients α and β are fitting terms provided by Jenkins et al. and which vary depending on the charge ratio of the salt.

### Solid-State Heat of formation: Co-Crystals

For lattice enthalpies calculated using PIXEL (Gavezzotti, 2005), calculations were set up using MrPIXEL (Reeves et al., 2020) within the Mercury interface, distributed with the Cambridge Structural Database (CSD) (Macrae et al., 2008; Groom et al., 2016). Hydrogen atom positions were set to the CSD normalised positions. For DFT-D, geometry optimisation calculations were performed using CASTEP17 (Clark et al., 2005) using the Perdew-Burke-Ernzerhof functional (Perdew et al., 1996) with a plane-wave basis set with a cut-off energy of 900 eV, which demonstrated convergence to 1 meV.atom^−1^. Norm-conserving pseudopotentials were used throughout, with a *k*-point spacing of 0.05 Å^−1^. The Tkatchenko-Scheffler dispersion correction scheme was applied. Lattice energies were determined by comparing the optimized energy values for the crystal structure with the energy for individual co-formers, modelled as effectively gas phase by removing all but one of the co-former molecules from the optimized crystal structure and computing a single point energy value using the same computational model as applied to the co-crystal. In cases where a unit cell vector was short, such that interactions with the nearest neighbour replica may occur (taken to be <5 Å), the smallest unit cell vector was doubled to ensure zero interaction. This was the case for **64**, **70** and **76**. Lattice enthalpies were then calculated using Eq. 9.

$$\Delta H_{L}=\frac{E_{cell}}{Z}-E_{co-former1}-E_{co-fomer2}$$ (9)

Where E_cell_ is the energy of the unit cell of the co-crystal and E_co-fomer1,2_ is the energy of each of the co-formers modelled in the “gas phase” and *Z* is the number of molecular units in the co-crystal. The ∆H_f(g)_ terms, to convert the ∆H_L_ terms to ∆H_f(s)_, were calculated using PM7 as documented above.

Lattice enthalpies of individual co-formers were calculated by adding the thermodynamic correction shown in Eq. 10 to the sublimation enthalpies calculated using the ESP method described above.

$$\Delta H_{L}=\;-\Delta H_{sub}-2RT$$ (10)

### Local Mode Force Constants

These were calculated using LMODEA (Kraka et al., 2020), following geometry optimisation and vibrational frequency calculation, for the 31 CHNO-containing molecules listed in Supplementary Table 6.

## Results and Discussion

### Calculating Gas Phase Heats of Formation

**FIGURE 1:**
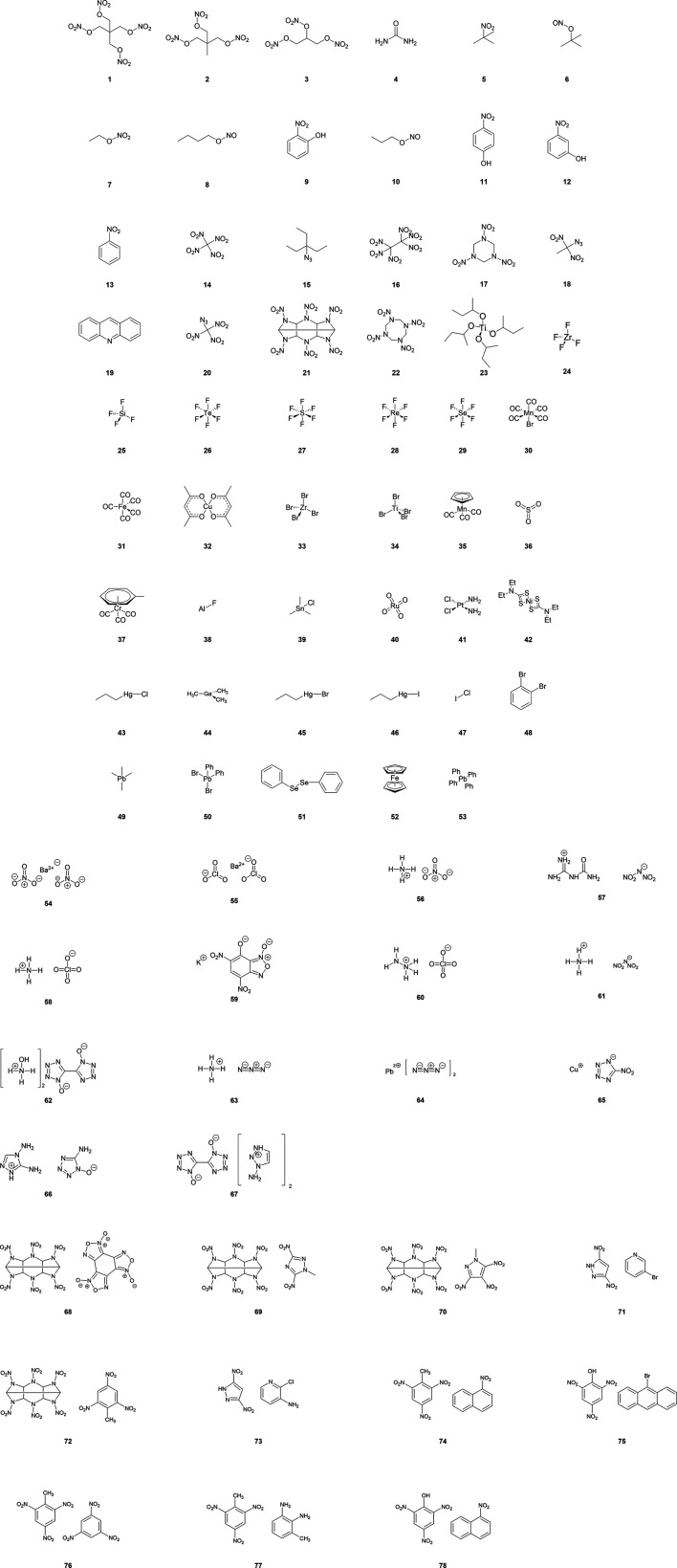
Molecules, salts and co-crystals employed in heat of formation energy calculations.

To test the three different methods for calculating ∆H_f(g)_ molecules **1**–**20** (see Figure 1 and Supplementary Table 1), were considered. Molecules were chosen as they had reliably reported experimental ∆H_f(g)_ available in the literature, and their less complex nature meant that the construction of isodesmic reaction equations was relatively straightforward (reported in full in Supplementary Table 2). As the PM7 method permits inorganic molecules to be studied, a further set of molecules which comprised main group and transition metal elements (**23**–**53**) was also studied. Notably, examples include molecules containing lead, copper and halogens, which are commonly found in EMs.

While the majority of CHNO-containing molecules investigated here have already been reported by Elioff et al. (2016), our process differs in that all molecules were first optimised at the 6-31G*/B3LYP level, followed by a single-point energy evaluation at PM7. In addition, the data set includes a further 4 molecules, namely **1, 2, 4,** and **19**, which incorporates the important EMs PETN (**1**), TMETN (**2**) into the test set. Calculation of ∆H_f(g)_ for the 31 inorganic molecules (**23–53**) by PM7 has not been reported before.

**FIGURE 2:**
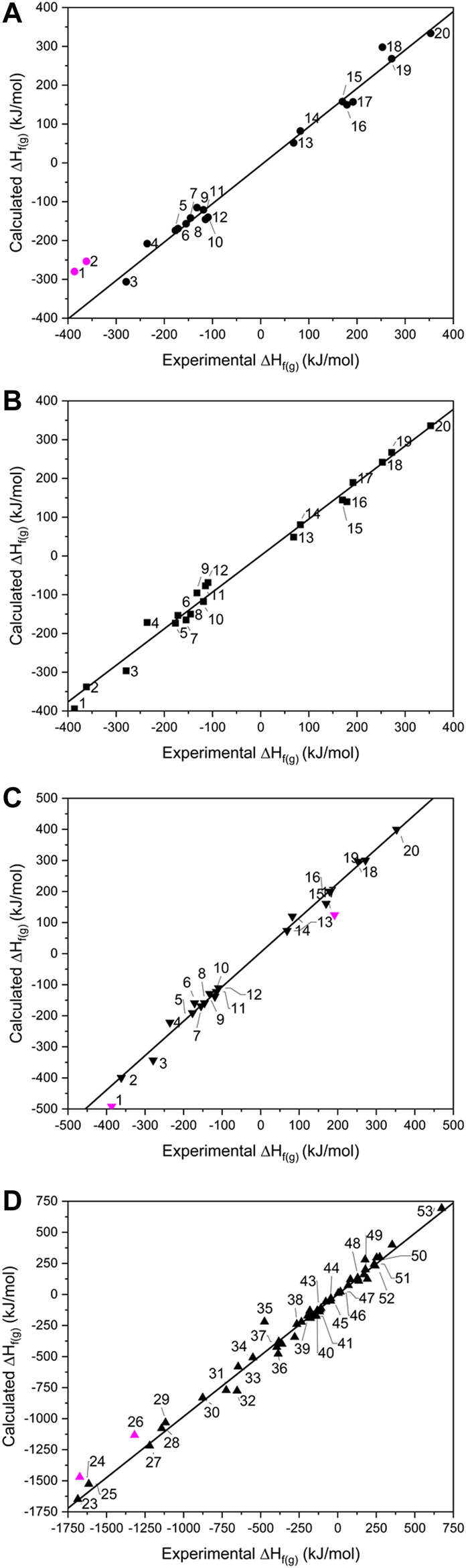
Showing calculated *vs.* experimental gas phase heats of formation for the **(A)** isodesmic, **(B)** atom equivalence, **(C)** PM7 for **1**–**20** and **(D)** PM7 for **23–53**. Data points omitted from the lines of best fit are shown in pink.

When calculated values of ∆H_f(g)_ are compared against experimental data (Figure 2), the two largest outliers for the isodesmic equation method (Figure 2A and Supplementary Table 3) were PETN and TMETN (**1** and **2** respectively), which deviate from the experimental values by 106.8 and 108.0 kJ mol^−1^, respectively. Both data points were disregarded when carrying out linear fitting, which otherwise returned an *R* ^2^ value of 0.994, and the gradient of the fitted line, *m* = 0.989. The reasons for failure for data points **1** and **2** must rest with either the experimental formation energies and/or the geometry optimisations of PETN and TMETN, or the geometry optimisation and/or experimental formation energy of another molecule defined in their respective isodesmic equations. The atom equivalence method (shown in Figure 2B) performs considerably better for these two compounds, which suggests that the experimental formation energies and the geometry optimizations, for both PETN and TMETN, are reliable. The isodesmic reactions constructed for these two molecules both include C(CH_3_)_4_, which is absent in all other isodesmic equations constructed for the test set (see Supplementary Table 2). Careful checking of the simulated geometry, to ensure the configuration obtained refers to the global minimum, suggests that the error most likely rests with the experimental heat of formation for C(CH_3_)_4_. This highlights a fundamental weakness with the isodesmic equation route, in that any error with any one term in the equation will render the calculated ∆H_f(g)_ for the target molecule as unreliable.

Using the atom equivalence method, no outliers where identified, showing the strength of this method for CHNO-containing molecules. This gave an *R* ^2^ value of 0.994 (*m* = 0.943), indicating that the atom equivalence method performs just as well as the isodesmic method.

Turning to the PM7 results (Figure 2C), PETN (**1**) again represents the largest deviation, with the semi-empirical method over-estimating the experimental value by 91.2 kJ mol^−1^. RDX (**17**) also appears to be less reliably calculated by this method, compared to the other two routes. Discarding **1** and **17** from the test set gives a line of best fit with *R* ^2^ = 0.993 (*m* = 1.083), suggesting overall that this method provides a similar level of accuracy compared to the isodesmic reaction and atom equivalence methods. The correlation with experimental data here is better than that reported by Elioff et al. (2016) (*R* ^2^ = 0.986) and considering the high overlap of molecules in test sets used, the improvement is likely due to the B3LYP/6-31G* geometry optimization step, as Elioff et al. relied on the PM7 method for structure optimisation as well as energy calculation. For the inorganic molecule list, **23–53,** which were tackled by PM7 only, the *R* ^2^ value increased slightly to 0.995, with the gradient of the line of best fit improved considerably [*m* = 0.983; omitting the largest outliers ZrF_4_ (**24**) and TeF_6_ (**26**), which deviate from experimental values by 186 and 203 kJ mol^−1^, respectively], suggesting its performance is just as reliable for inorganic molecules as it is for organic molecules. It is unclear why ZrF_4_ and TeF_6_ deviate so much from the expected values. Other related compounds, **25**, **27–29** and **34**, have been calculated accurately (although **26** is the only Te-containing compound and Zr is represented just twice in the data set). The possibility of experimental inaccuracy also cannot be ruled out.

### Calculating Solid Heats of Formation

**FIGURE 3:**
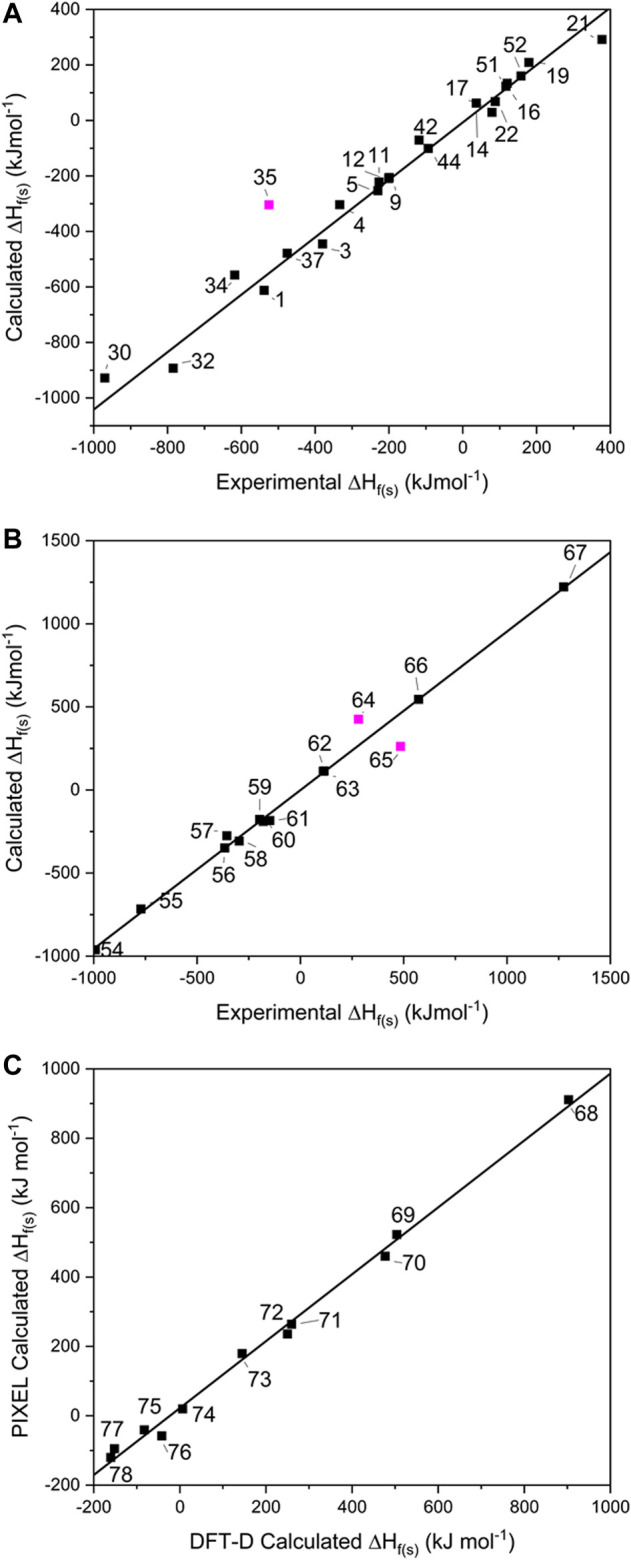
Showing the calculated solid heats of formation for **(A)** single component crystals, **(B)** salts and **(C)** co-crystals. Data points omitted from the lines of best fit are shown in pink.

To probe the conversion of ∆H_f(g)_ to ∆H_f(s)_, 23 of the single-component EMs were selected from the **1**–**53** test set for which experimentally determined ∆H_f(s)_ were available (see Figure 3A and Supplementary Table 1), and further pursued using the ESP method. This includes 14 CHNO-containing molecules and 9 inorganic compounds. Fourteen salts (**54**–**67** in Figure 1) were similarly pursued using Jenkins’ method, while 11 co-crystals (**68**–**78** in Figure 1) were explored using PIXEL and DFT-D.

For the single-component crystals, ∆H_f(s)_ calculated using the ESP method offered a line of best fit of *R* ^*2*^ = 0.995, *m* = 1.100 [omitting one point that lay off the line, Mn(CO)_3_Cp (**35**) by 220 kJ mol^−1^, see Figure 3A], indicating a good predictive result has been obtained across a broad spectrum of compounds and broad range of energies.

For the EM salts (**54**–**67**), application of Jenkins’ method gives a line of best fit through the simulated values (see Figure 3B) with *R* ^2^ = 0.997 (*m* = 0.955), and a maximum deviation from experimental values of 80.5 kJ mol^−1^ for FOX-12 (**55**). This is an improvement in the correlation reported by Gao et al. (2007) who reported an *R* ^*2*^ of 0.984 for a set of 33 inorganic and CHNO EM salts, of which 19 were inorganic and the remainder CHNO. While acknowledging that the improved result obtained here could be due to the smaller test set employed in this work, it could also indicate that the PM7 method, used to calculate the ∆H_f(g)_ terms for the constituent ions, offered an improvement over the approach adopted by Gao, who relied on isodesmic equations. This latter approach is likely to be particularly problematic here as experimental formation energies of ions are required. Our data set shares two data points with Gao’s (salts **56** and **61**); closer inspection of these predictions shows that both ∆H_f(s)_ values were calculated more accurately here, with **56** deviating from the experimental values by 17 kJ mol^−1^ (Gao’s prediction differed by 24.7 kJ mol^−1^), whereas **61** deviated by only 0.9 kJ mol^−1^ (Gao by 63.5 kJ mol^−1^). This would therefore appear to support further the use of PM7 to calculate the ∆H_f(g)_ terms. There are two outliers in this fitting – lead azide (**64**) and DBX-1 (**65**), which deviate from their literature values by 222 and 145 kJ mol^−1^, respectively. We note that both exist as extended coordination complexes in the solid state, which may contribute towards their poor prediction by Jenkins’ method, which was formally devised for salts.

It should be noted that TKX-50 (**62**) has two values for ∆H_f(s)_ reported in the literature. The most widely reported is 446.6 kJ mol^−1^ (Sabatini and Oyler, 2015), which was the value calculated using Jenkins’ method, with the ∆H_f(g)_ values for the constituent ions calculated using the CBS-4M atomisation method. However, Sinditskii et al. (2015) have argued that this value is questionable when compared to the sum of the enthalpies of formation for the individual components of TKX-50 and when compared with typical heats of reaction between acids and bases. They performed bomb calorimetry experiments and determined ∆H_f(s)_ to be 111 ± 16 kJ mol^−1^, far lower than the widely reported calculated value. Our computed value, also derived from the Jenkins’ method, but utilising PM7 to calculate the ∆H_f(g)_ values for the ions, is 112.6 kJ mol^−1^, showing that the earlier result was in error due to computation of the ∆H_f(g)_ terms for the molecular ions.

For the 11 energetic co-crystals investigated, ∆H_L_ was calculated using two different approaches, the quicker PIXEL method, and the more computationally demanding DFT-D method (see Figure 3C and Supplementary Table 6), as no experimental data was available to benchmark the predictions against. From these results, it was readily apparent that the two methods provide comparable results (*R* ^2^ = 0.997, *m* = 0.964), meaning that both options are viable for co-crystals as part of a computational screening program. PIXEL does present some limitations, however, as it is not applicable to structures where the number of molecules in the crystallographic asymmetric unit exceeds 2; for these larger crystal lattices DFT-D is at present the only realistic solution.

**FIGURE 4:**
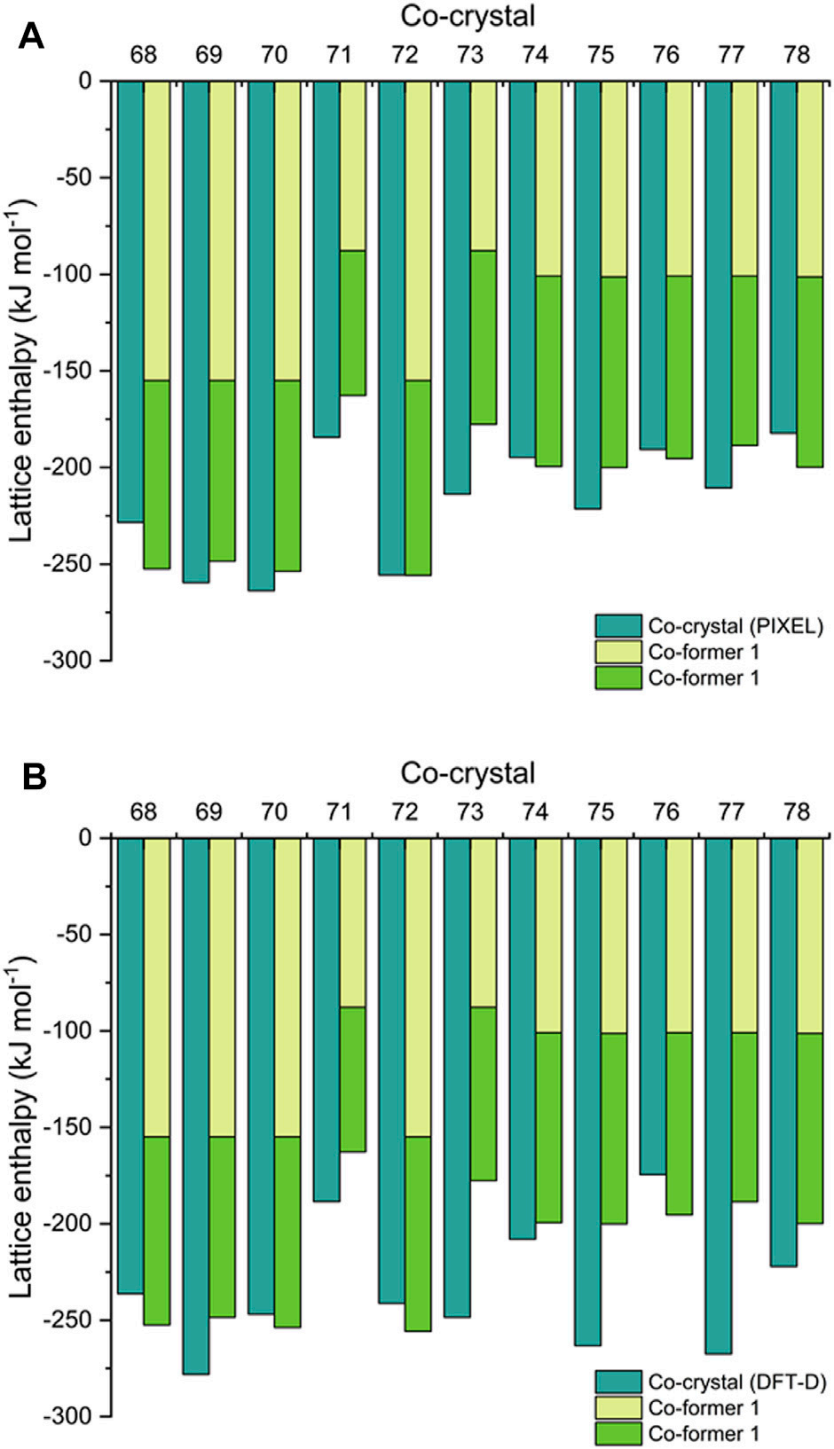
Comparison of calculated lattice enthalpies of co-crystals **68**–**78** by **(A)** PIXEL and **(B)** DFT-D, alongside constituent co-formers.

Next, we offer an interesting comparison between the ∆H_L_ of the co-crystals (from Eq. 9) alongside the ∆H_L_ values for their respective co-formers (from Eqs. 5, 10). This is shown in Figure 4 (and Supplementary Tables 1, 6), from which it is apparent that the sum of the latter gives a reasonable approximation of the former. This is in agreement with Vener et al., who studied a number of co-crystals using DFT-D and hybrid-DFT functionals (Vener et al., 2014). A similar observation was also made by Day et al., who showed using DFT-D simulations that the total energy of 350 organic co-crystals was greater than a linear weighed sum of their single-component counterparts by the order of just *ca*. 8 kJ mol^−1^ (per molecule). This applied to around 95% of their data set, showing that co-crystal formation is overwhelmingly driven by thermodynamics (Taylor and Day, 2018). Estimating ∆H_L_ for a co-crystal by this quick approximation may help guide the choice of co-formers for EM research, in order to maximize the amount of stored chemical energy. It also would be particularly useful to estimate ∆H_L_ for co-crystals such as CL-20/HMX, which has a 2:1 ratio of co-formers (Bolton et al., 2012) and thus falls outside the scope of PIXEL calculations. Accordingly, we estimate ∆H_L_ of CL-20/HMX to be *ca*. −430 kJ mol^−1^ [based on ∆H_L_ (HMX) + 2 × ∆H_L_ (CL-20), see Supplementary Table 1]. Using DFT-D, the corresponding ∆H_L_ prediction is -460 kJ mol^−1^. This results in a predicted ∆H_f(s)_ of *ca*. 570–600 kJ mol^−1^. We note that Zhang et al. (2019) estimated a considerably higher value for ∆H_f(s)_ of 861.9 ± 18.6 kJ mol^−1^; this was obtained indirectly using a calculated heat of reaction for the formation of solid CL-20/HMX from solid ε-CL-20 and β-HMX, that in turn used a thermochemical cycle based on measured enthalpies of dissolution of all species in acetonitrile, and literature ∆H_f(s)_ values for ε-CL-20 and β-HMX.

While Day et al. makes a strong case for co-crystallisation being overwhelmingly thermodynamically driven (Taylor and Day, 2018), a recent report by Perlovich suggests that the formation of around 30% of co-crystals are entropically driven (Perlovich, 2020). This work was based on constructing a dataset of 1947 co-crystals for which experimental sublimation energies were available, from which an algorithm to calculate the Gibbs sublimation energy was derived. Our test set of eleven EM co-crystals is too small to add any significant weight to this argument. While in general the predictions show that the lattice energy of the co-crystal does indeed exceed that of the sum of the co-formers (by up to *ca.* 70 kJ mol^−1^, a similar total energy range reported by Day et al.), for some structures (**68, 74, 76** and **78** by PIXEL, **68**, **72** and **76** by DFT-D) the relationship does not hold, although we note that the energy shortfalls are typically very small. Improving the computational method to improve the accuracy of the comparison (*i.e.,* zero point energy and entropy corrections) (Nyman and Day, 2015) will be considered more broadly in follow up studies. Such corrections could be obtained from calculating full phonon spectra, but these are time consuming to perform and therefore go against the computational screening philosophy which pervades this paper.

In summary, of the three methods tested here for calculating ∆H_f(g)_, PM7 performs favourably compared with the isodesmic reactions and atom equivalence methods, while offering application to the widest range of molecules. Its strength has also been demonstrated when calculating ∆H_f(s)_ for single component solids and salts, by the ESP and Jenkins methods, respectively, with predicted values showing excellent correlation with experimental ∆H_f(s)_ values. Finally, two different methods, PIXEL and DFT-D, were compared for calculating ∆H_L_ terms for co-crystals and found to give comparable results. Comparison of the calculated ∆H_L_ terms show that, to a first approximation, the lattice enthalpy of the co-crystal is the sum of the lattice enthalpies of the constituent co-formers. While this does not inform on the likelihood of success for the formation of new co-crystals, it does offer significant new insight in directing co-crystallisation studies to create new EMs with desired ∆H_f(s)_ values.

### Local Force-Constant Calculations

**FIGURE 5:**
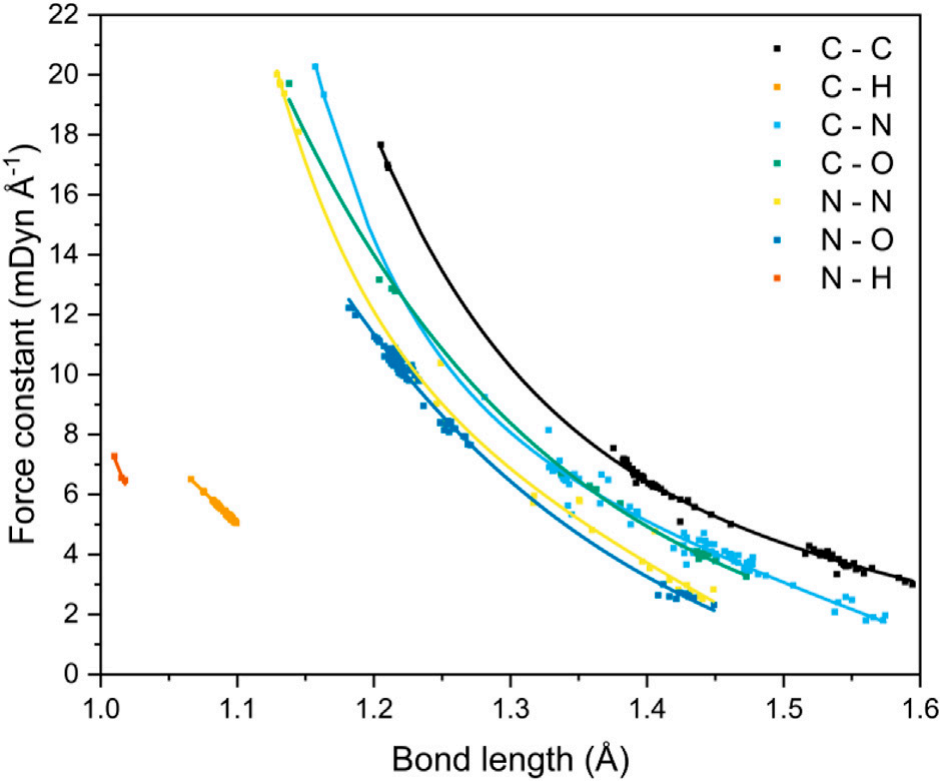
Correlation between bond lengths and local vibrational mode force constants for seven covalent bonding environments.

Local vibrational-mode force constants were calculated for all CHNO-containing molecules in the test set, with a further ten EMs added to provide a wider and more comprehensive coverage of bond length values (31 CHNO molecules in total, Supplementary Table 7, including the EMs CL-20, RDX, HMX, HNB, NTO, TATB, FOX-7, PETN and nitroglycerin). From this data, a relationship between the bond lengths and force constants (bond strengths) of seven different covalent bonding environments can be drawn (see Figure 5).

The data shown here expands upon the relationship that has been established by Kraka et al. for C−C bonds occurring in both gas and solid state geometries.(Kraka et al., 2020). It also mirrors the trends shown by Byler et al. (1987) and Ladd et al. (1964) for C−N bonds and C−O bonds, respectively. The studies by Byler and Ladd analyzed mainly linear molecules in an effort to reduce the effect of coupling of normal vibrational modes. This limitation is elegantly side-stepped by local vibrational mode analysis through the recasting of the normal modes of vibration into the local modes through mass decoupling (Groom et al., 2016; Hehre et al., 1970) Byler et al*.* reported bond lengths that ranged from 1.122 Å (*k* = 20.17 mdyn Å^−1^, H_3_CC−NBCl_3_) to 1.555 Å (*k* = 3.51 mdyn Å^−1^, F_3_C−NO). Their data sit comfortably on our C−N curve, highlighting the power of the local-mode analysis route for obtaining force constants for more complex molecules. Ladd et al*.* measured the vibrational frequency of the C−O bond stretch for different excited states of carbon monoxide to obtain the relationship between force constant and bond length over the range 1.088–1.396 Å. Their data agree quantitatively with the C–O bond curve shown in Figure 5 at long bond lengths, but consistently underestimates the force constants at shorter (<1.2 Å) distances, which suggests that measuring the force constants of only excited states of CO has skewed the relationship between bond length and force constant. McKean made extensive studies of the vibrational frequencies of isolated C–H bond stretches, a property which is directly comparable to the local mode of vibration (McKean, 1978; Konkoli et al., 1998; Konkoli and Cremer, 1998a; Larsson and Cremer, 1999), and therefore the force constants, and presented a relationship between C–H bond length and experimentally determined stretching frequencies which mirrors the trend shown here. Cremer et al. (2000) also calculated C–H force constants of adiabatic internal modes (an earlier name for local vibrational modes), their shortest C–H bond being 1.086 Å with a force constant of 5.58 mdyn Å^−1^. This fits on our line presented in Figure 5, which is now further extended to 1.066 Å (and 6.46 mdyn Å^−1^).

A strong correlation of force constant and bond length is observed for all bond types, and decay functions were used to fit trend lines (Supplementary Table 8) that returned *R* ^2^ values of *ca.* 0.99 for all bond types, with the exception of C−N (*R* ^2^ = 0.98) and N−H (*R* ^2^ = 0.92), although the latter corresponds to only four data points and is likely under-represented. Four bond types, C−C, C−N, C−O and N−N, encompass single to triple bond behaviour, and N−O bond types include single and double bonds, as evident from the clustering of data points. The wide range of bond lengths studied give rise to a corresponding wide range of calculated local-force constant values, which for the most part follow the sequence C−C > C−N ≈ C−O > N−N ≈ N−O, although the longer distances associated with single C−N bonds render these interactions on a par with single N−N and N−O bonds. The weakness of the long C–N bond fits with expectation: the rupture of R–NO_2_ bonds has been shown to be a critical step in the decomposition of energetic materials (Sharma and Owens, 1979; Sharma et al., 1982; Owens, 1996). The data shown in Figure 5 are testament to the great potential offered by the local-mode analysis route: it is a quick and straightforward method to compare and contrast the bond strengths of all bonding interactions within a molecule, from which the weakest bond can be unambiguously identified. In essence, the curves shown in Figure 5 allow a direct mapping between bond length and bond strength to be obtained. For the case of CL-20, a cage nitramine structure comprising 5, 6 and 7 membered C−N rings (compound **21** in Figure 1), it has been suggested that the C−N bonds forming the cage can also act as the “trigger linkage,” (Sun et al., 2021) in addition to the generally weaker N−NO_2_ bonds. This is supported here, with the C−N bonds in the more strained 5-membered rings having force constants similar to the stronger N−NO_2_ bonds (3.337 *vs.* 3.151 mdyn Å^−1^, respectively). The less strained 6-membered ring contains stronger C−N bonds, with force constants ranging from 4.088–4.486 mdyn Å^−1^, a considerable increase in strength from the C−N bonds present in the 5-membered rings. The relationship between bond length and force constant presented here therefore has the potential to be applied to molecular design, as the weakest bond in a molecule can be tuned by its surrounding molecular environment.

## Data Availability

The original contributions presented in the study are included in the article/Supplementary Material, further inquiries can be directed to the corresponding author.
